# A Rapid and Surfactant-Free Synthesis Strategy for Variously Faceted Cuprous Oxide Polyhedra

**DOI:** 10.3390/nano15030240

**Published:** 2025-02-04

**Authors:** Kaihao Liu, Yu Xin, Shikun Gao, Yadong Yu, Mengyan Dai, Zhe Liu

**Affiliations:** 1 Chemical Defense Institute, Academy of Military Sciences, Beijing 102205, China; kyle197@163.com (K.L.); xinyu_0619@163.com (Y.X.); 323085404204@stu.suse.edu.cn (S.G.); yuyadong36@163.com (Y.Y.); 2 Taiyuan Satellite Launch Center, Taiyuan 030032, China; 3 School of Automation and Information Engineering, Sichuan University of Science and Engineering, Yibin 644000, China

**Keywords:** cuprous oxide, surfactant-free, polyhedral morphology, wet-chemical reduction method, micro/nanocrystals

## Abstract

We systematically investigated the morphology-controlled synthesis of Cu_2_O micro-nano crystals, especially under surfactant-free conditions, targeting a simple, rapid, and morphologically controllable preparation strategy for polyhedral Cu_2_O micro-nano crystals. By systematically investigating the effects of NaOH concentration, types of reducing agents, and copper salt precursors on crystal growth, precise control over the morphology of Cu_2_O crystals under surfactant-free conditions was achieved. This method can rapidly prepare variously faceted Cu_2_O crystals under mild conditions (70 °C, 7 min), including regular polyhedra with low-index facets exposure including cubes, octahedra and rhombic dodecahedra, as well as more complex polyhedra with high-index facets exposure such as 18-faceted, 26-faceted, 50-faceted and 74-faceted crystals. NaOH concentration is found to be the key factor in controlling Cu_2_O crystal morphology: as the concentration of NaOH increases, the morphology of Cu_2_O crystals gradually transforms from cubes that fully expose the {100} faces to regular polyhedra that expose the {110}, {111} faces, and even other high-index faces, ultimately presenting octahedra that fully expose the {111} faces. Additionally, Cu_2_O crystals with unique morphologies such as hollow cubes and 18-faceted with {110} face etched can be obtained by introducing surfactants or prolonging reaction durations. This work provides new insights into the morphology control of Cu_2_O crystals and establishes foundation in acquiring distinct Cu_2_O polyhedra in a facile manner for their application in catalysis, optoelectronics, sensing, and energy conversion fields.

## 1. Introduction

In recent decades, the morphology-controlled synthesis of micro/nanocrystals has been a research hotspot in the field of materials science [1,2,3]. The morphology and surface conditions (surface energy and electronic structure) of inorganic micro/nanocrystals have a significant impact on their physical and chemical properties. Different shapes of micro/nanocrystals expose different Miller-indices facets with distinct atomic arrangements and charge density states on the surfaces, which leads to differences in chemical activity, optoelectronic properties and so forth [4,5].

As a p-type semiconducting material, cuprous oxide (Cu_2_O) has attracted considerable attention due to its unique properties such as a direct bandgap of 2.17 eV, the earth abundance of copper element, its environmental friendliness, and its low cost [6]. The customizable morphology and the resulting special properties have enabled a wide range of applications of Cu_2_O in areas like energy conversion, catalysis, sensing, and template-directed nanostructure synthesis [7,8,9,10]. The performance of Cu_2_O-based materials is closely related to their crystal morphology, exposed facets, and surface structures. Cu_2_O belongs to the cubic crystal system (space group *Pn$\bar{3}$m*), where each Cu atom is linearly coordinated to two O atoms, and each O atom is tetrahedrally coordinated to four Cu atoms [11,12]. The surface atomic arrangements and electronic structures vary significantly among different crystal facets, and these unique surface properties normally result in different chemical reactivities [5,13,14]. For example, differences in surfaces state such as dangling bonds and lone pairs of valance electrons cause selectivity disparity among Cu_2_O octahedra, cubes, and rhombic dodecahedra in catalyzing propylene oxidation reaction with O_2_. Cu_2_O octahedra exposing {111} crystal planes are believed to be more selective for acrolein. In contrast, Cu_2_O cubes exposing {100} crystal planes are more selective for CO_2_, while Cu_2_O rhombic dodecahedra with {110} crystal planes are more favorable for propylene oxide. One-coordinated Cu on Cu_2_O(111), three-coordinated O on Cu_2_O(110), and two-coordinated O on Cu_2_O(100) were identified as the catalytically active sites for the production of acrolein, propylene oxide, and CO_2_, respectively [15].

Previous studies indicate that electrochemical deposition [16,17], chemical vapor deposition [18,19], and magnetron sputtering [20,21] are suitable to prepare Cu_2_O thin films, while hydrothermal methods [22,23] or wet-chemical reduction methods [24,25] are widely adopted for the synthesis of Cu_2_O polyhedra. By meticulously manipulating the reaction process, the wet-chemical reduction method has flexible and controllable capabilities in adjusting the nucleation and growth rates along different directions [26,27]. This advantage has made the wet-chemical method widely accepted in controlling the exposed facets and size distribution of Cu_2_O micro/nanocrystals [28,29,30]. However, most of the reported wet-chemical reduction methods for the synthesis of Cu_2_O polyhedra rely on the use of surfactants to control the exposure of specific facets, which may introduce surface contamination and affect the intrinsic properties of Cu_2_O. For instance, Zhang used polyvinylpyrrolidone (PVP) to control the exposure area of the {111} facets [24]. For the synthesis of polyhedra exposing high-index facets, the complex combination of surfactants or longer reaction times are usually required. For example, Wang et al. used sodium dodecyl sulfate (SDS) as a surfactant to control the synthesis of concave octahedra to expose the {322} facets [10]. Leng et al. used different organic solvents to control the exposure of various high-index facets [31]. Jiao et al. synthesized the 74-faced polyhedra with exposed {211} and {744} facets in a relatively long reaction scheme (over 60 min) [32]. Kim et al. synthesized several Cu_2_O polyhedra, ranging from cubes to octahedra, under surfactant-free conditions, but they did not prepare Cu_2_O polyhedra exposing high-index facets [11]. In surfactant-free synthesis methods reported by others, the processes typically yield Cu_2_O crystals of a single morphology, or require significantly different procedures to obtain different morphologies, making rapid switching between morphologies difficult [12]. So, it is necessary to develop a surfactant-free, rapid, and simple method for controllable synthesis of Cu_2_O micro-nano crystals with various morphologies.

**Figure 1:**
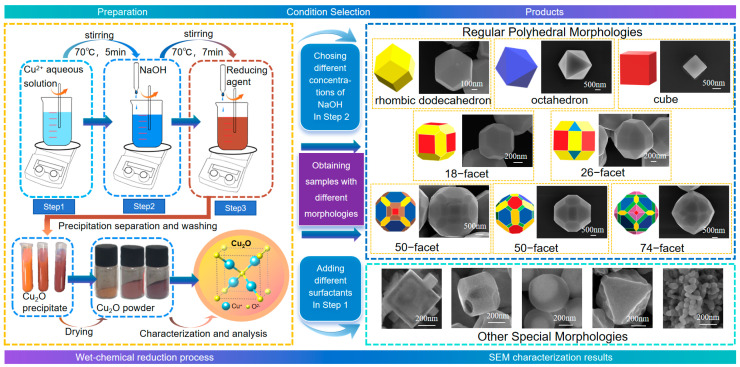
A schematic flowchart for the controllable and rapid preparation of surfactant-free Cu_2_O polyhedral crystals.

Based on the growth mechanism of Cu_2_O crystals, this study proposes a new morphology control strategy by precisely tuning the OH^−^ concentration in the reaction system. It was found that the OH^−^ concentration not only affects the type of copper precursor during reaction, but also significantly changes the growth rates of different crystal facets, thereby enabling the controllable regulation of crystal morphology. Beyond this aspect, we further compared the effects of various reducing agents and copper precursors. As shown in Figure 1, this method requires only a simple adjustment of the reaction precursors, without using any surfactant, can rapidly (within 7 min at 70 °C) produce a variety of Cu_2_O polyhedra, including cubes, octahedra, and rhombic dodecahedra with single set of low-index facets exposed, as well as 18-faceted, 26-faceted, 50-faceted, and 74-faceted with multiple index facets exposed. Furthermore, by using dilute NaOH, lower reaction temperature or introducing specific surfactants, core–shell, hollow, etched and other unique structures of Cu_2_O crystals can also be obtained.

## 2. Experimental Section

### 2.1. Chemicals

The chemicals used include copper(II) sulfate pentahydrate (CuSO_4_·5H_2_O, 99.995%, Aldrich, USA), copper(II) chloride dihydrate (CuCl_2_·2H_2_O, ≥99%, Aldrich, USA), sodium hydroxide (NaOH, 97%, Macklin, China), D-(+)-glucose (C_6_H_12_O_6_, ≥99%, Sigma, USA), L-ascorbic acid (AA,C_6_H_8_O_6_, ≥99%, Aldrich, USA), hydroxylamine hydrochloride (NH_2_OH·HCl, ≥99%, Aldrich, USA), oleic acid (C_18_H_34_O_2_, 90%, Aldrich, USA), sodium dodecyl sulfate (SDS, C_12_H_25_SO_3_Na, ≥99%, Aldrich, USA), ethanol (C_2_H_6_O, ≥99.8%, Macklin, China), and cyclohexane (C_6_H_12_, ≥99%, Macklin, China). All solutions were prepared using deionized water (18.2 MΩ·cm at 25 °C).

### 2.2. Characterization

The crystal structure and phase purity of the as-prepared Cu_2_O powder samples were characterized by X-ray powder diffraction (Bruker D8 Advance, Germany) using Cu Kα radiation (λ = 1.5418 Å) at a scan rate of 1 °/min in the 2θ range of 10–80°. The morphology and size of the Cu_2_O samples were examined by scanning electron microscopy (TESCAN MIRA LMS, Czech) at accelerating voltages of 3–20 kV. Transmission electron microscopy (JEOL JEM-F200, Japan) was used to obtain transmission electron (TEM), high-resolution transmission electron (HRTEM), and selected area electron diffraction (SAED) images at an accelerating voltage of 200 kV.

### 2.3. Rapid Synthesis of Morphology-Controlled Cu_2_O Polyhedra Without Surfactants

The Cu_2_O micro/nanocrystal samples were synthesized through a simple wet chemical reduction procedure in a total volume of 10 mL. Taking the synthesis of Cu_2_O cubes as an example, 0.2497 g (1.0 mmol) of CuSO_4_·5H_2_O was first dissolved in 8.5 mL of deionized water and heated to 70 °C under vigorous stirring. Then, 0.5 mL of 10 M NaOH aqueous solution was added dropwise. After stirring for 5 min at 70 °C, 1.0 mL of 0.7 M D-(+)-glucose aqueous solution was added, and the reaction was maintained at 70 °C for another 7 min. The resulting brick-red precipitate was collected by centrifugation at 6000 rpm for 5 min, washed with deionized water and ethanol for at least three times, respectively. All centrifugation and washing steps in this study were performed using the same centrifuge (Kecheng H3-18K, China, g = 9.8015 m/s^2^). The product was finally dried at 50 °C under vacuum for 24 h to obtain the Cu_2_O powder sample.

To prepare Cu_2_O crystals with different morphologies, multiple experiments were performed by varying the concentration of NaOH. To further elucidate the shape evolution trend of Cu_2_O, different types of copper sources and reducing agents were also employed. As a result, we obtained Cu_2_O polyhedra with excellent morphology and uniformity, including rhombic dodecahedra, cubes, and octahedra that expose a single set of low-index crystal facets; 18-faceted and 26-faceted polyhedral structures that expose two or more sets of low-index faces; and polyhedra with high-index facets exposed such as 50-faceted and 74-faceted polyhedra. The corresponding reaction conditions and morphologies are shown in Table 1.

### 2.4. Synthesis of Other Morphologies of Cu_2_O Micro/Nanocrystals

**Hollow cubic Cu_2_O:** An amount of 0.1705 g (1.0 mmol) of CuCl_2_·2H_2_O was dissolved in 8.8 mL of deionized water and heated to 70 °C under vigorous stirring. Then, 0.2 mL of 10 M NaOH aqueous solution was added dropwise. After stirring for 5 min at 70 °C, 1.0 mL of 0.1 M AA aqueous solution was added, and the reaction was maintained at 70 °C for another 7 min. The resulting precipitate was separated from the solution by centrifugation at 6000 rpm for 5 min, washed several times, and after drying, hollow Cu_2_O cubes were obtained.

**Eighteen-faceted Cu_2_O microcrystals with the {110} facets etched:** Quantities of 0.1705 g (1.0 mmol) of CuCl_2_·2H_2_O and 0.174 g of SDS were dispersed in 8.7 mL of deionized water and stirring at room temperature. Then, 0.1 mL of 1.0 M NaOH aqueous solution was added dropwise, and the mixture was stirred for 10 min. Subsequently, 1.2 mL of 0.1 M NH_2_OH·HCl aqueous solution was added. The reaction vial was shaken vigorously for 20 s, and then aged at room temperature for 1 h. The color of the mixture gradually turned orange; finally, the resulting precipitate was separated from the solution by centrifugation at 6000 rpm for 5 min. After washing several times and drying, etched {110} facet Cu_2_O 18-faceted microcrystals were obtained.

**Aggregates of Sub-50 nm Cu_2_O nanoparticles:** Quantities of 0.1705 g (1.0 mmol) of CuCl_2_·2H_2_O and 0.174 g of SDS were dispersed in 8.6 mL of deionized water and mixture was stirred at room temperature until it became homogeneous solution. Then, 0.2 mL of 2.0 M NaOH aqueous solution was added dropwise, and the mixture was stirred for 10 min. Subsequently, 1.2 mL of 0.1 M AA aqueous solution was added. The reaction vial was shaken vigorously for 20 s and then aged at room temperature for 1 h. The color of the mixture gradually turned brick red. Finally, the resulting precipitate was separated from the solution by centrifugation at 6000 rpm for 5 min. After washing several times and drying, aggregates of sub-50 nm Cu_2_O nanoparticles were obtained.

**Facet-etched octahedral and spherical core–shell structures of Cu_2_O crystals: An amount of** 0.1705 g (1.0 mmol) of CuCl_2_·2H_2_O was dispersed in 4.0 mL of deionized water, and then 0.25 mL of oleic acid and 2.0 mL of anhydrous ethanol were added under vigorous stirring. When the mixture was heated to 100 °C, 1.0 mL of 0.8 M NaOH aqueous solution was added. After stirring for 5 min, 3.0 mL of 0.23 M D-(+)-glucose solution was added, and the reaction was allowed to proceed for 60 min when the color of the reactants gradually changed to brick red. Finally, the resulting precipitate was separated from the solution by centrifugation at 6000 rpm for 5 min. The precipitated product after separation is washed with hexane and anhydrous ethanol for more than three times each. After drying, facet-etched octahedral were obtained. Using the same reaction conditions but maintaining the temperature at 50–60 °C, core–shell structured Cu_2_O crystals could be obtained.

## 3. Results and Discussion

Herein, we employed a bottom-up wet chemical synthesis strategy to prepare Cu_2_O micro/nano-crystals with various morphologies. The fundamental chemical principle of reducing Cu^2+^ to Cu^+^ is similar to that of the Fehling’s reaction: in the alkaline environment, the Cu^2+^ ions form blue Cu(OH)_2_ precipitate (Equation (1)), which further reacts with OH^−^ to generate the [Cu(OH)_4_]^2−^ complex ions (Equation (2)). The [Cu(OH)_4_]^2−^ complexes are then reduced by the reducing agents (e.g., D-(+)-glucose) to form the brick-red Cu_2_O precipitates (Equation (3)).

(1) $${Cu}^{2+}+2{OH}^{-}\to {Cu(OH)}_{2}↓$$

(2) $${Cu(OH)}_{2}+2{OH}^{-}\to {{\left[Cu(OH\right)}_{4}]}^{2-}$$

(3) $${{2[Cu(OH)}_{4}]}^{2-}+C_{5}H_{11}O_{5}-CHO\to {Cu}_{2}O↓+C_{5}H_{11}O_{5}-COOH+4{OH}^{-}+2H_{2}O$$

According to the Gibbs–Wulff theory, under thermodynamic equilibrium conditions, the equilibrium crystal morphology is determined by minimizing the total surface free energy of the system, and the facets with lower surface energy are more likely to be exposed. The surface energy of Cu_2_O follows the order of γ_{111}_ < γ_{100}_ < γ_{110}_, so the most common Cu_2_O morphologies are octahedra and cubes [33]. Rhombic dodecahedra are typically prepared under weakly acidic conditions, which is believed to be advantageous for lowering the surface energy of the {110} facets [7]. Hence, we chose NH_2_OH·HCl as the reducing agent to regulate the pH and prepared rhombic dodecahedra form.

In light of the aforementioned principles, the morphology control of Cu_2_O polyhedra can be achieved by simply changing the NaOH concentration without any surfactant. Under varying NaOH concentrations, by appropriately replacing the reducing agent and copper salt precursor, polyhedral Cu_2_O with excellent crystallinity and smooth facets can be obtained.

### 3.1. Regular Polyhedral Morphologies of Cu_2_O

**Figure 2:**
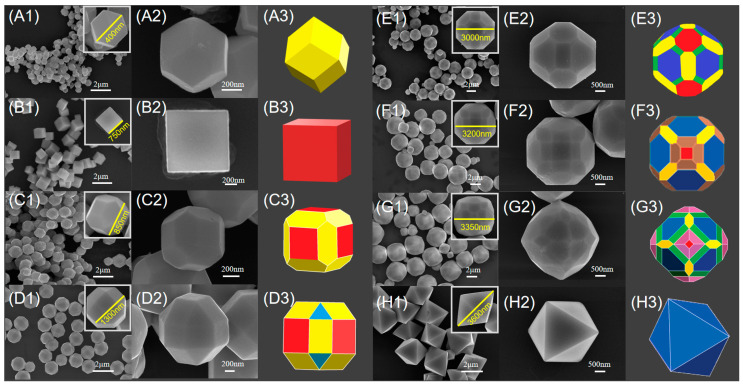
The letters represent: (**A**) rhombic dodecahedra, (**B**) cubes, (**C**) 18−faceted polyhedra, (**D**) 26−faceted polyhedra, (**E**) 50−faceted polyhedra, (**F**) 50−faceted polyhedra, (**G**) 74−faceted polyhedra, (**H**) octahedra. The numbers represent: (1) Low-magnification SEM images, (2) high-magnification SEM images, (3) simulated structures of the Cu_2_O crystals. The facets in the simulated structures of the Cu_2_O crystals have the following colors: yellow for {110} facets, red for {100} facets, blue for {111} facets, green for {211} facets, brown for {522} facets, and purple for {744} facets.

Various types of Cu_2_O polyhedra were synthesized under the reaction conditions listed in Table 1, and their SEM images are shown in Figure 2. Using CuSO_4_ solution as the Cu^2+^ precursor and 0.24 M NH_2_OH·HCl as the reducing agent, with the NaOH concentration controlled at 0.3 M, Cu_2_O rhombic dodecahedra with ~400 nm in diameter and exposing twelve {110} facets were obtained (Figure 2A1–3). Changing the reducing agent to 0.07 M glucose solution and adjusting the NaOH concentration to 0.5 M, uniform cubic Cu_2_O crystals with ~750 nm in diameter and exposing six {100} facets were obtained (Figure 2B1–3). Using 0.07 M AA as the reducing agent and adjusting the NaOH concentration to 1.0 M, 18-faceted Cu_2_O crystals with~850 nm in diameter and composed of {100} and {110} facets were obtained (Figure 2C1–3), The 18-faceted polyhedra are formed by truncating twelve edges of cubes. Using an aqueous solution of CuCl_2_ as a Cu^2+^ precursor, and maintaining the NaOH concentration at 1.0 M, 26-faceted Cu_2_O crystals with~1300 nm in diameter and exposing {100}, {110} and {111} low-index facets were obtained (Figure 2D1–3). Using 0.07 M glucose as the reducing agent and increasing the NaOH concentration to 1.2 M, 50-faceted Cu_2_O crystals with ~3000 nm in diameter and exposing high-index {211} facets were obtained (Figure 2E1–3). Replacing the reducing agent with 0.07 M AA and increasing the NaOH concentration to 1.2 M, 50-faceted Cu_2_O crystals with ~3200 nm in diameter and exposing high-index {522} facets were obtained (Figure 2F1–3). Using CuCl_2_ and AA, with the NaOH concentration controlled at 1.4 M, 74-faceted Cu_2_O crystals with ~3350 nm in diameter were obtained. This complex polyhedron is composed of 6 square {100} facets, 8 hexagonal {111} facets, 12 hexagonal {110} facets, 24 high-index rectangular {211} facets and 24 high-index trapezoidal {744} facets (Figure 2G1–3). Further increasing the NaOH concentration to 3.0 M, the small-area facets on the Cu_2_O polyhedra disappeared, and octahedral Cu_2_O crystals with ~3600 nm in diameter and fully exposing {111} facets were obtained (Figure 2H1–3). These Cu_2_O crystals with different morphologies were all prepared without the use of any surfactant.

From the perspective of crystal facets with different Miller indices, the low-index facets {100}, {110}, and {111} have larger relative areas and are more readily formed. On the contrary, higher surface energy leads to smaller areas and less stability for high-index facets, such as {211}, {522}, and {744}. Moreover, these high-index facets with smaller areas are typically rougher than the low-index facets, indicating slightly poorer crystallinity and a higher defect density. Such defects are often positively associated with the catalytic activity of Cu_2_O.

**Figure 3:**
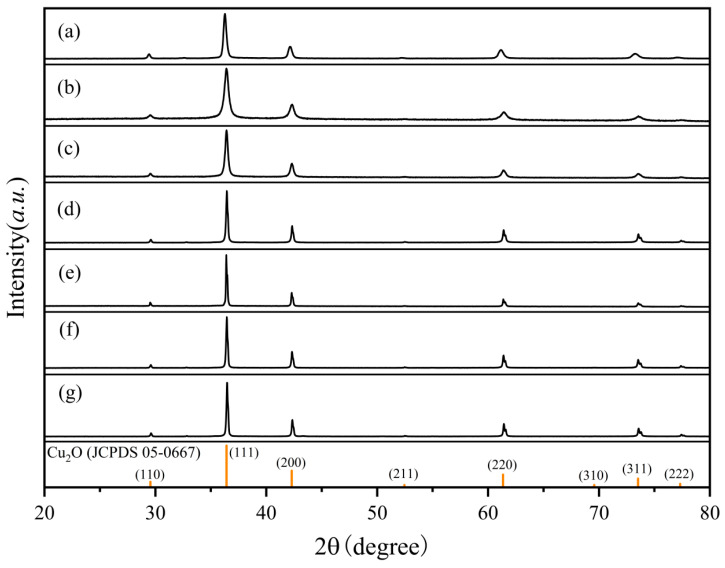
XRD patterns of Cu_2_O powder samples with different morphologies: (**a**) rhombic dodecahedra, (**b**) cubes, (**c**) 18-faceted polyhedra, (**d**) 26-faceted polyhedra, (**e**) 50-faceted polyhedra, (**f**) 74-faceted polyhedra, (**g**) octahedra.

The XRD patterns of the different morphology Cu_2_O powder samples (Figure 3) all match well with the standard cubic phase of Cu_2_O (JCPDS 05-0667, a = 0.4269 nm). The positions and the relative intensities of the peaks in the spectra for Cu_2_O of different morphologies are essentially the same, but the peak widths vary. According to the Scherrer Equation (4):

(4) D=Kλ/(βcosθ)

K is a constant, D represents the grain size, λ denotes the wavelength of the X-rays, β is the full width at half-maximum (FWHM) or the integrated width (IW) of the diffraction peak, and θ is the angle of diffraction. The smaller the crystal size of the sample, the broader the peak width. The rhombic dodecahedra and cubic crystals synthesized are smaller in size, which results in broader diffraction peaks in the spectrum. In contrast, the polyhedra and octahedra exposing high-index facets are larger in size, leading to narrower diffraction peaks. This is consistent with the conclusions drawn from the SEM images.

**Figure 4:**
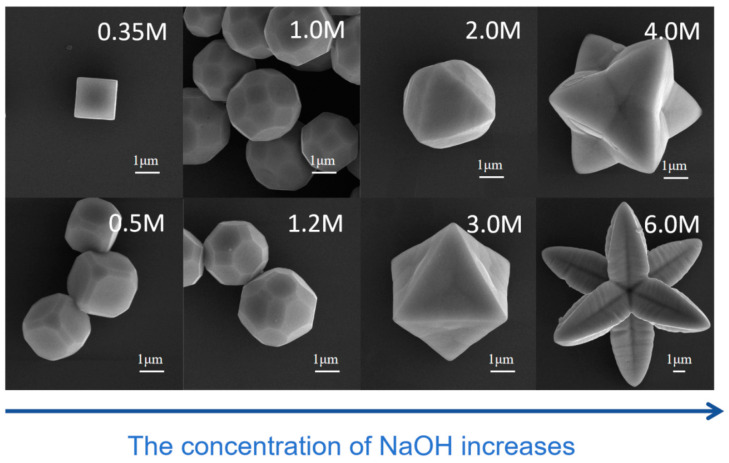
SEM images of Cu_2_O prepared under the same reaction conditions with different concentrations of NaOH. The NaOH concentration is indicated in the top right corner.

Through the results of preparation experiments of Cu_2_O crystals with different morphologies, it can be seen that the Cu_2_O crystals synthesized under higher NaOH concentrations often have larger crystallite sizes. To verify this conclusion, with the concentrations of CuCl_2_ and D-(+)-glucose fixed at 0.1 M and 0.07 M, respectively, and the reaction temperature at 70 °C and reaction time of reduction at 7 min, different Cu_2_O crystal samples were prepared by simply adjusting the NaOH concentration. As shown in Figure 4, when the NaOH concentration was 0.35 M, the edge length of the cubic Cu_2_O was about 1.5 μm, and as the NaOH concentration gradually increased, the size of the Cu_2_O crystals continued to increase, reaching a diameter of 6.5 μm for the concave octahedral Cu_2_O micro-crystals at an NaOH concentration of 4.0 M and 12 μm for the “petal-like” Cu_2_O at an NaOH concentration of 6.0 M.

The influence of NaOH on the shape evolution of Cu_2_O crystals was verified. As shown in Figure 4, in the alkaline environment, the lower the NaOH concentration, the greater the inhibition of the growth rate of the {111} facet, and the more easily cubic Cu_2_O crystals are formed. As the NaOH concentration increases, the growth rates along the six spatial coordinate directions (<100>) of the crystals increase significantly, the exposed area of the {100} facet decreases, and the exposed area of the {111} facet increases, forming octahedra at a certain concentration (around 3.0 M); as the concentration further increases, the product morphology changes to concave octahedra and eventually petal-like structures. Typically, the {100} facet surface is covered by O atoms, the {110} facet surface covered by both Cu and O atoms in a bidentate configuration, while the {111} facet surface is covered by singly coordinated Cu atoms. Therefore, the {100} and {110} surfaces are electrically neutral, while the {111} surface is positively charged [13]. As the concentration of OH^−^ increases, the concentration of [Cu(OH)_4_]^2−^ ions also increases, and they preferentially bind to the positively charged {111} facets. Consequently, the surface energy of the {111} faces are reduced, leading to the easier exposure of the {111} facets at higher NaOH concentrations, and thus the easier formation of octahedron morphology [11].

**Figure 5:**
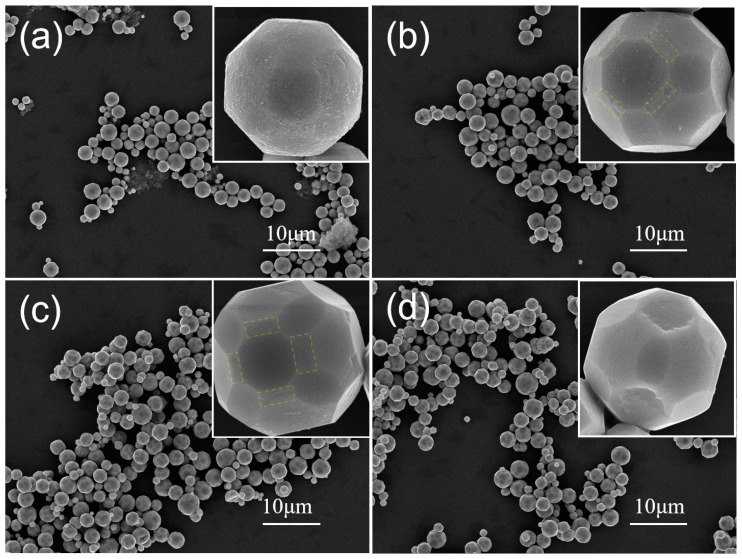
SEM images of Cu_2_O obtained with the same reaction conditions but different reaction times of (**a**) 1, (**b**) 4, (**c**) 7, and (**d**) 12 min. The yellow dashed area in the figure represents the high-index {211} facets.

To disclose the formation mechanism of Cu_2_O microcrystals, we studied the morphological evolution process of 50-faceted Cu_2_O crystals under various reaction durations (Figure 5). At the beginning of the reaction within 1 min, the sample had already taken on a quasi-50-faceted shape, but the surfaces are rather rough (Figure 5a). It is believed that this morphology transformation process of Cu_2_O crystals is ascribed to the classical ion-mediated growth process (ripening) [29]. Compared to the product within 1 min of the reduction reaction, the product obtained after 4 min of the reduction reaction shows a marked improvement in crystallinity. High-index {211} facets began to appear, but they had a smaller area and a rough surface. (Figure 5b). When the reduction is prolonged to 7 min, the degree of surface roughness of 50-faceted Cu_2_O crystals decreases, the high-index {211} facets are more distinct, and crystals with smooth surfaces and sharp edges are obtained (Figure 5c). When the reaction time was further extended to 12 min, the high-index small facets of the 50-faceted polyhedra disappeared with the {100} facets recessed, and eventually evolved into concave 26-faceted polyhedra (Figure 5d).

Based on the evolution process observed over different periods of reduction time, it can be speculated that a large number of Cu_2_O nanocrystallites first nucleate and grow into small seed particles. To minimize the overall energy of the system, these small seed particles tend to aggregate together and directly form quasi-50-faceted morphologies with rough surfaces instead of undergoing a transformation from other Wulff shapes. Simultaneously, owing to the ripening mechanism, the quasi-multifaceted structures gradually transform into perfect 50-faceted polyhedra. As the reaction time further increases, high-index facets with high surface energy gradually diminish and eventually disappear, while the {100} facets, which have the lowest surface energy, become recessed due to their slow growth rate.

**Figure 6:**
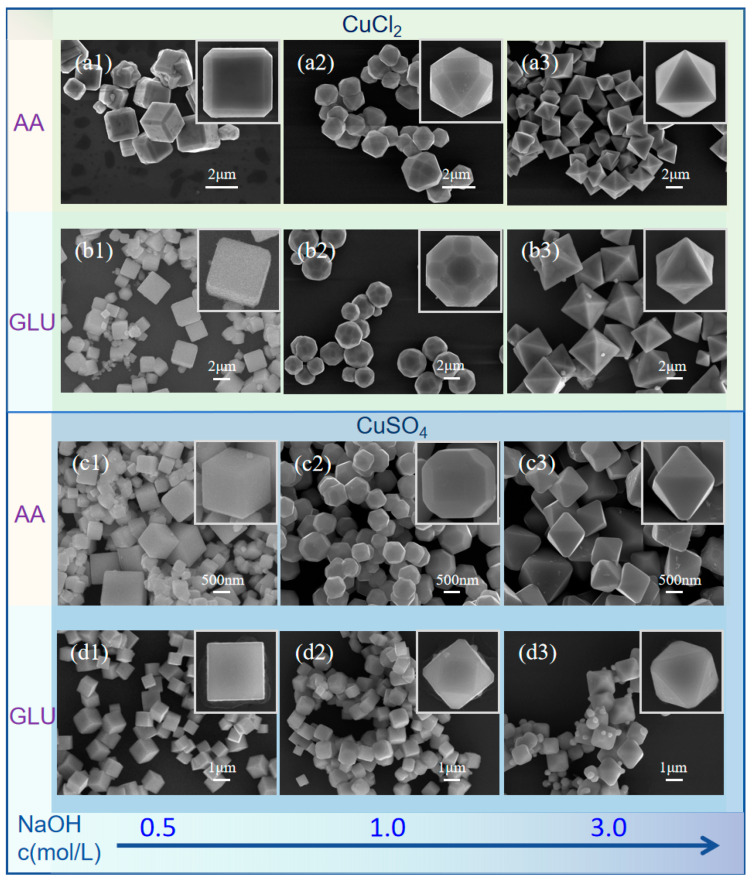
SEM images of Cu_2_O crystal morphologies prepared under different conditions: In the figures, GLU stands for D-(+)-glucose, (**a**–**d**) indicate different combinations of reaction precursors: (**a**) CuCl_2_+AA, (**b**) CuCl_2_+GLU, (**c**) CuSO_4_+AA, (**d**) CuSO_4_+GLU. (1–3) indicate different concentrations of NaOH: (1) 0.5M, (2) 1.0M, (3) 3.0M.

The type of reducing agent also affects the morphology of Cu_2_O (Figure 6). With low NaOH concentration (0.5 M), AA may result in truncated edges and corners, forming a mixture of Cu_2_O 26-faceted polyhedra out of the cubic Cu_2_O sample (Figure 6a1). By contrast, using D-(+)-glucose as the reducing agent will produce cubic product with clearer crystal edges and better uniformity (Figure 6b1,d1). Under medium NaOH concentration (1.0 M) conditions with AA as the reducing agent, the products obtained with CuCl_2_ and CuSO_4_ as the Cu^2+^ precursors are 26-faceted (Figure 6a2) and 18-faceted (Figure 6c2) Cu_2_O, respectively. When the reducing agent is changed to D-(+)-glucose, the products obtained with CuCl_2_ and CuSO_4_ as the Cu^2+^ precursors are 50-faceted (Figure 6b2) and 14-faceted (Figure 6d2) Cu_2_O, respectively. For the preparation of octahedra under high NaOH concentrations (3.0M), the products prepared from AA have better crystallinity with clearer facet boundaries and crystal edges compared to those of D-(+)-glucose (Figure 6a3,c3). It is apparent that the latter often result in concavity of the {111} facets and the presence of irregular Cu_2_O nanoparticles (Figure 6b3,d3). This may be attributed to the fact that D-(+)-glucose uses the aldehyde group in the molecule to reduce Cu^2+^ to Cu^+^ and the aldehyde group is unstable in the strong alkaline environment. On the contrary, AA has stronger reducing ability due to the multiple hydroxyl groups and enol structure in the molecule. Therefore, in the strong alkaline environment, the morphology regulation effect of glucose is not as good as that of AA.

Due to the ab-/adsorption effects on the surface of Cu_2_O crystals, different anions present in the reaction process have certain influence on the final morphology of Cu_2_O. Figure 6 shows that both CuSO_4_ and CuCl_2_ aqueous solution as Cu^2+^ precursors can be adopted to prepare cubic Cu_2_O crystals at low NaOH concentration (around 0.5 M) and prepare octahedral Cu_2_O crystals at relatively high NaOH concentration (around 3.0 M). However, at low NaOH concentration, the cubic Cu_2_O crystals prepared using CuSO_4_ aqueous solution as the precursor (Figure 6c1,d1) have smoother surfaces and sharper edges than using CuCl_2_ aqueous solution as the precursor (Figure 6a1,b1). At medium NaOH concentration, the Cu_2_O crystals prepared using CuCl_2_ are more likely to expose more varieties of Miller index facets (Figure 6a2,b2). At high NaOH concentration, the octahedral Cu_2_O crystals prepared using CuCl_2_ have better uniformity and the crystal edges are more blunt. (Figure 6a3,b3). Generally speaking, Cu_2_O crystals prepared using CuCl_2_ are larger in size than those prepared using CuSO_4_.

Despite the difference in starting materials, the shape evolution of Cu_2_O follows similar pattern. Cubic or cuboid shapes of Cu_2_O are prepared at low concentration of OH^−^ and the shape gradually approaching octahedra as the concentration of OH^−^ increases coupled with the increase in crystallite size. Although the reducing agent and the type of anions have certain impact on the morphology of Cu_2_O, the key factor influencing the pattern of morphological evolution hinges on the concentration of OH^−^.

### 3.2. Other Special Morphologies of Cu_2_O Micro/Nanocrystals

When using dilute NaOH solution or introducing SDS, oleic acid and other capping agents into the reaction system, some unique morphologies of Cu_2_O crystals can be achieved.

**Figure 7:**
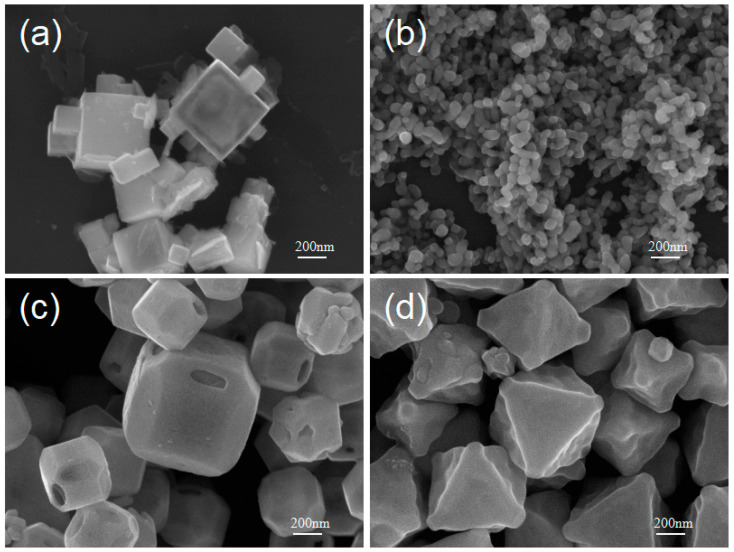
SEM images of special morphologies of Cu_2_O micro/nanocrystals: (**a**) hollow cubes, (**b**) agglomerates of sub-50 nm Cu_2_O nanoparticles, (**c**) 18-faceted microcrystals with the {110} facets etched, (**d**) facet-etched octahedral.

As shown in Figure 7a, when the molar ratio of CuCl_2_ to AA in the reaction precursors is 1:0.7 and the NaOH concentration is 0.2 M, the product shows partially hollow cubic structures. This is possibly due to the fact that under relatively low c[OH^−^], the concentration of [Cu(OH)_4_]^2−^ in the solution is very low and the growth rate of Cu_2_O crystals decreases. A slower growth rate is beneficial for the self-assembly of primary Cu_2_O nanoparticles into porous nanocubes. This distinct cubic structure is presumed to be formed through an oriented attachment mechanism by which Cu_2_O nanocrystallites bind in an orderly manner, preserving the overall cubic symmetry of the crystals. The presence of inter-crystallite space in the Cu_2_O aggregates facilitates the hollowing process while the dissolved Cu^2+^ ions are reduced to Cu_2_O and preferentially deposited on the surface crystal surface leading to the formation of this hollow structure [34]. If SDS is introduced as a capping agent in the system, and the molar ratio of NaOH to CuCl_2_ to AA is 1:0.25:0.3, nano-sized Cu_2_O aggregates can be obtained. As shown in Figure 7b, these aggregates are composed of many Cu_2_O nanoparticles less than 50 nm in diameter. SDS exhibits a specific adsorption effect on the surface of Cu_2_O crystals, thus changing the binding energy of the Cu_2_O crystal surface. The addition of a large amount of SDS can effectively limit the size of Cu_2_O crystals, while other surfactants such as PVP or CTAB do not have such a pronounced effect on size control. We have tested PVP at amounts ranging from 0.005 g to 0.7 g and CTAB at amounts from 0.125 g to 0.3 g as surfactants in our experiments. The results indicated that PVP and CTAB were not effective in controlling the morphology and size of Cu_2_O crystals, but they both effectively prevent nanoparticle aggregation. As shown in Figure 7c, we used NH_2_OH·HCl as the reducing agent and SDS as the capping agent. By carefully adjusting the molar ratio of the starting materials and prolonging the reaction time, 18-faceted Cu_2_O hollow shells with {110} facets etched can be obtained. The formation of this morphology is possibly due to the specific adsorption effect of SDS on the {110} facets of the Cu_2_O crystals coupled with the etching effect of Cl^−^ released from NH_2_OH·HCl during reaction [35]. The etched {110} facets expose new highly active site beneficial for catalysis.

**Figure 8:**
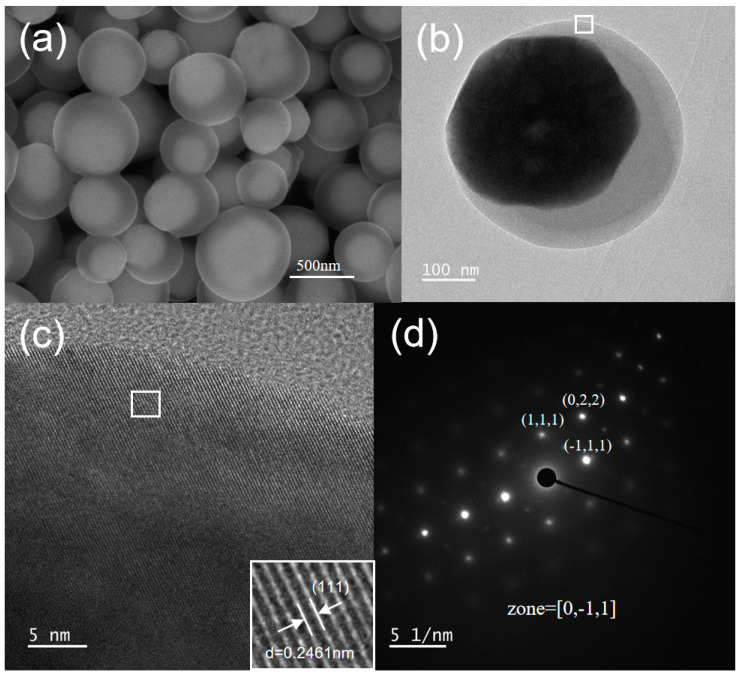
The core–shell Cu_2_O crystals prepared under oleic acid-ethanol conditions: (**a**) SEM image, (**b**) TEM image, (**c**) HRTEM image, (**d**) SEAD image.

There is a strong interaction between the surface of Cu_2_O crystals and oleic acid molecules [29]. When ethanol and oleic acid are introduced into the reaction system and the reaction is carried out at 100 °C with addition of 0.25 mL of oleic acid, a new type of Cu_2_O octahedral-like crystal structure is obtained, as shown in Figure 7d. This octahedral-like crystal has a rough surface, which may have superior catalytic performance over smooth surfaces. In the oleic acid-ethanol reaction system, when the reaction temperature is lowered to 50 °C, core–shell structured Cu_2_O crystals can be obtained. Figure 8a shows the SEM image of the core–shell structured Cu_2_O prepared under the oleic acid-ethanol conditions. This core–shell structure is composed of the polyhedra or small spherical structured Cu_2_O in the interior and a spherical thin film on the outer layer. Through TEM and HRTEM analysis of the outer shell structure (Figure 8b,c), the lattice fringe spacing of the outer shell is d = 0.2461 nm, which matches well with the (111) plane spacings (d = 0.2465 nm) in the cubic phase Cu_2_O crystal (JCPDS 05-0667). The SAED image (Figure 8d) also indicates that the outer shell layer is a well-crystallized Cu_2_O single crystal.

## 4. Conclusions

This study used a simple wet-chemical method to successfully synthesize various regular Cu_2_O polyhedra, including cubes, octahedra, rhombic dodecahedra, 18-faceted, 26-faceted, 50-faceted, and 74-faceted polyhedra, as well as unique concave polyhedra, convex polyhedra, and petal-like structures, without the addition of any capping agents. The results show that the concentration of NaOH in the reaction process plays a crucial role in the morphology control of the Cu_2_O products. Under weakly acidic conditions, the Cu_2_O product presents as rhombic dodecahedra fully exposing the {110} facets. At low NaOH concentrations, the Cu_2_O product is mainly cubic with {100} facets exposed. As the NaOH concentration increases, the Cu_2_O exhibits combinations of multiple Miller index facets. At specific NaOH concentrations, high-index facets can also be exposed leading to the formation of 18-faceted, 26-faceted, 50-faceted, and 74-faceted polyhedra. With a further increase in the NaOH concentration, the morphology gradually transforms from convex octahedra to octahedra, concave octahedra, and finally, petal-like structures.

By introducing different surfactants such as SDS or oleic acid, unique Cu_2_O crystal morphologies can also be obtained, including hollow cubes, 18-faceted microcrystals with the {110} facets etched, aggregates of sub-50 nm Cu_2_O nanoparticles, octahedral with surface imperfections, and core–shell structures.

The morphology of Cu_2_O products is also affected by reaction temperature, though controlling these conditions is more complex. Systematic experiments have not yet been conducted in this study. Future studies could explore the principles of Cu_2_O morphology control by regulating reaction temperature under various conditions.

It is foreseeable that by designing Cu_2_O material from the perspective of crystal morphology control combined with doping, ion exchange, heterojunction, and superlattice structures, more novel Cu_2_O-based material system can be constructed. Such a material engineering strategy will greatly expand the application prospects of Cu_2_O in the fields of optoelectronics, sensing, and energy conversion.

## Figures and Tables

**Table 1:** Cu_2_O morphologies corresponding to different reaction conditions.

Sample	Copper Precursor	NaOH Concentration	Reducing Agent	Morphologies	Exposed Miller Index Facets	Average Sizes
A	CuSO_4_ 0.1M	0.3M	NH_2_OH·HCl 0.24M	rhombic dodecahedra	12{110}	400 nm
B	CuSO_4_ 0.1M	0.5M	D-(+)-glucose 0.07M	cubes	6{100}	750 nm
C	CuSO_4_ 0.1M	1.0M	AA 0.07M	18-faceted polyhedra	6{100}&12{110}	750 nm
D	CuCl_2_ 0.1M	1.0M	AA 0.07M	26-faceted polyhedra	6{100}&12{110}&8{111}	1300 nm
E	CuCl_2_ 0.1M	1.2M	AA 0.07M	50-faceted polyhedra	6{100}&12{110}&8{111} &24{211}	3000 nm
F	CuCl_2_ 0.1M	1.2M	D-(+)-glucose 0.07M	50-faceted polyhedra	6{100}&12{110}&8{111} &24{522}	3200 nm
G	CuCl_2_ 0.1M	1.4M	AA 0.07M	74-faceted polyhedra	6{100}&12{110}&8{111} &24{211}&24{744}	3350 nm
H	CuCl_2_ 0.1M	3.0M	AA 0.07M	octahedra	8{111}	3600 nm

## Data Availability

The original contributions presented in the study are included in the article, further inquiries can be directed to the corresponding authors.

## Abbreviations

The following abbreviations are used in this manuscript:

SDS	sodium dodecyl sulfate
PVP	polyvinylpyrrolidone
AA	L-ascorbic acid
GLU	D-(+)-glucose
CTAB	cetyltrimethylammonium bromide
